# UHPLC-HRMS-Based Untargeted Lipidomics Reveal Mechanism of Antifungal Activity of Carvacrol against *Aspergillus flavus*

**DOI:** 10.3390/foods11010093

**Published:** 2021-12-30

**Authors:** Chenling Qu, Zhuozhen Li, Xiupin Wang

**Affiliations:** 1 Grain and Oil Storage Department, College of Food Science and Engineering, Henan University of Technology, Zhengzhou 450001, China; 201992034@stu.haut.edu.cn; 2 Oil Crops Research Institute, Chinese Academy of Agricultural Sciences, Wuhan 430062, China

**Keywords:** *Aspergillus flavus*, carvacrol, untargeted lipidomics, ultra-high-performance liquid chromatography-high-resolution mass spectrometry (UHPLC-HRMS), antifungal activity

## Abstract

*Aspergillus flavus* is a common contaminant in grain, oil and their products. Its metabolite aflatoxin B_1_ (AFB_1_) has been proved to be highly carcinogenic. Therefore, it is of great importance to find possible antifungal substances to inhibit the growth and toxin production of *Aspergillus flavus*. Carvacrol (CV) was reported as a potent antifungal monoterpene derived from plants. In this paper, the antifungal effects and mechanism of CV on *Aspergillus flavus* were investigated. CV was shown good inhibition on the growth of *Aspergillus flavus* and the production of AFB_1_. CV used in concentrations ranging from 0, 50, 100 and 200 μg/mL inhibited the germination of spores, mycelia growth and AFB_1_ production dose-dependently. To explore the antifungal mechanism of CV on *Aspergillus flavus*, we also detected the ergosterol content of *Aspergillus flavus* mycelia, employed Scanning Electron Microscopy (SEM) to observe mycelia morphology and utilized Ultra-High-Performance Liquid Chromatography-High-Resolution Mass Spectrometry (UHPLC-HRMS) to explore the lipidome profiles of *Aspergillus flavus*. The results showed that the production of ergosterol of mycelia was reduced as the CV treatment concentration increased. SEM photographs demonstrated a rough surface and a reduction in the thickness of hyphae in *Aspergillus flavus* treated with CV (200 µg/mL). In positive ion mode, 21 lipids of *Aspergillus flavus* mycelium were downregulated, and 11 lipids were upregulated after treatment with 200-µg/mL CV. In negative ion mode, nine lipids of *Aspergillus flavus* mycelium were downregulated, and seven lipids upregulated after treatment with 200-µg/mL CV. In addition, the analysis of different lipid metabolic pathways between the control and 200-µg/mL CV-treated groups demonstrated that glycerophospholipid metabolism was the most enriched pathway related to CV treatment.

## 1. Introduction

As a pathogenic fungus, *Aspergillus flavus* Link (*A. flavus*) frequently contaminates grains, oil and nuts, resulting in a significant reduction of nutritional quality and a serious loss of food commodities [1,2]. Since *A. flavus* could produce hazardous secondary metabolite aflatoxins (AFTs), it also causes contamination of food commodities during storage and processing and endangers human health [3]. AFTs mainly includes aflatoxin B_1_ (AFB_1_), aflatoxin B_2_ (AFB_2_), aflatoxin G_1_ (AFG_1_) and aflatoxin G_2_ (AFG_2_) [4]. Among them, AFB_1_ is considered to be the most mutagenic and teratogenic and is well-documented to be a pathogenic agent of hepatocellular carcinoma [5]. Therefore, it is of great importance to control aflatoxin contamination.

Using synthetic preservatives and fungicides was an effective way to inhibit the growth of *A. flavus* and the production of AFTs. However, the application of synthetic antifungal agents had led to a series of problems, such as drug resistances, and was harmful to human health [6,7]. Consequently, investigators had been searching for new natural products to control *A. flavus* reproduction and to reduce AFTs contamination.

EOs are natural aromatic oily liquids obtained from flowers, leaves, roots, bark, fruits, seeds and resin [8]. A number of studies had reported the essential oils (EOs) were effective substances with antifungal activity and had been kept under Generally Recognized as Safe (GRAS) category by United States Food and Drug Administration [9]. Previous reports demonstrated that EOs performed antifungal activity by the way of destruction of fungal plasma membrane of fungus [10,11]. Oxidative stress, membrane permeability and amino acid metabolism were significantly changed after perilla frutescens essential oil-treated *A. flavus* [12]. The essential oil from dill (*Anethum graveolens* L.) caused morphological changes in the cells of *A. flavus* and a reduction in the ergosterol quantity [13]. Thymol has a variety of antifungal mechanism of action, such as disruption of cell membranes, inhibition of efflux pumps, alteration of mycelial morphology and production of ROS and nitric oxide [14].

As one kind of EO compounds, Carvacrol (CV, C_10_H_14_O), 5-isopropyl-2-methylphenol, is a liquid phenolic monoterpenoid found in herb like oregano (*Origanum vulgare*), pepperwort (*Lepidium flavum*), wild bergamot (*Citrus aurantium var. Bergamia Loisel*) and other plants [15,16]. Hsin-Bai Yin [17] investigated the effect of CV on contaminated poultry feed and discovered that CV can reduce AFTs production in broth culture and chicken feed by at least 60% when compared to control group, downregulated the expression of main genes (*aflC, nor1* and *norA*) associated with AFT synthesis in the molds. Sharifi et al. [18] presented that the antimicrobial potential bioactivities of CV were high due to the presence of a free hydroxyl group, hydrophobicity and a phenol moiety. For a more comprehensive analysis of the antifungal mechanism of CV, a lipidomics analysis of *A. flavus* may provide important information.

Lipidomics, which aimed to study the lipid of specific tissues or organisms at the system-wide level, was widely used in food science for a variety of purposes [19]. Lipids are one of the main classes of compounds in biological systems and perform important physiological tasks. Their hydrophobicity allows them to form cell membranes that constitute a boundary against the hydrophilic environment of the cells [20]. As the important components of biological membranes, lipids (e.g., phospholipids, sphingolipids and phytosterol) are crucial for their pathogenicity and could be a potential target to inhibit fungal growth [21,22]. In recent years, some studies on the effect of EOs on the lipid profile of *A. flavus* were reported. Helal et al. found that mycelia fumigated with *Cymbopogon citratus* L. essential oil reduced the total lipid content, reduced the saturated fatty acid and increased the unsaturated fatty acid compared to samples without EO treatment [23]. Paeonol acting on *A. flavus* reduced the total lipid content, induced lipid peroxidation and altered the glycerol content [24].

As of today, the underlying antifungal mechanism of CV on *A. flavus* is still far from unequivocal. Therefore, in the present study, we evaluated the inhibitory of CV on the growth and toxin production of *A. flavus*. The UPLC-HRMS-based untargeted lipidomics approach was utilized for lipid profile analysis in *A. flavus*. A multivariate statistical analysis was used to analyze significant lipids and metabolic pathways to clarify alterations in lipid metabolic pathways of *A. flavus* treated by CV. Our work mainly explored the potential anti-*A. flavus* mechanism of CV so as to provide a theoretical reference for the use of CV for control of *A. flavus*.

## 2. Materials and Methods

### 2.1. Materials

The strain *A. flavus* 127-1 was isolated from the soil of the principal peanut regions (Liaoning Province, China) and preserved in our laboratory, which was a medium aflatoxin producer. CV was purchased from Jiangxi Cedar natural medicinal oil Co., Ltd. (Ji an, China). All other chemicals were analytical grade. Formic acid and ammonium formate were obtained from Aladdin Biochemical Technology Co., Ltd. (Shanghai, China). Deionized water was purified by a Milli-Q system (Millipore-Co., Ltd., Milford, MA, USA) and was used to prepare all aqueous solutions. Dichloran Glycerol-18 Agar medium (DG-18) and Liquid Sabourand medium were purchased from Haibo Biotechnology Co., Ltd. (Shanghai, China).

### 2.2. Fungal Culture

The strain was cultured in DG 18 Agar medium (Haibo Biotechnology Co., Ltd.) (Qingdao, China) at a constant temperature and humidity (28 °C, 75%) for 5 days. The *A. flavus* spores were washed off by 0.1% Tween-80 solution, and *A. flavus* spore suspension with 2 × 10^8^ spores/mL was obtained.

### 2.3. Inhibitory Effect of CV on A. flavus Growth and Toxin Production

#### 2.3.1. Inhibitory Effect of CV on Spore Germination

Twenty microliters of *A. flavus* spore suspension (2 × 10^8^ spores/mL) were inoculated into 20-mL liquid sabourand medium with different concentrations of CV (0, 50, 100 and 200 μg/mL) and cultured at 28 °C for 8 h. Then, the spore germination was observed by a light microscope. When the length of the germination tube was longer than the diameter of the spore, the spore was considered as “germinated”.

#### 2.3.2. Inhibitory Effect of CV on *A. flavus* Mycelia Growth

The antifungal activity of CV was estimated by the contact agar phase effects on the mycelia growth of *A. flavus*. Primarily, 500 µL of CV was well-dissolved in 4.5 mL of 0.1% Tween-80. Different amounts of CV solution were aseptically transferred into disposable plastic petri dishes containing 20 mL of preheated DG18 agar under sterile conditions, respectively. The sample without CV treatment was used as the control. After the DG 18 agar medium was colloid, 5 µL of *A. flavus* spore suspension was added at each culture plate. *A. flavus* was continuously cultured at 28 °C and 75% humidity for 5 days, and the colony diameter was measured by the crossing method every day [25].

#### 2.3.3. Inhibitory Effect of CV on Mycelia Dry Weight

A series of volumes of CV was pipetted aseptically into a conical flask containing 50 mL of the liquid sabourand medium to procure the required concentrations of CV 50, 100 and 200 μg/mL. The sample without CV treatment was used as the control. One hundred microliters of *A. flavus* spore suspension (2 × 10^8^ spores/mL) were cultured at each conical flask. After incubating at 28 °C and a speed of 200 r/min for 5 days, the *A. flavus* mycelia solution was obtained. Then, the mycelia were collected, lyophilized and weighed.

#### 2.3.4. Inhibition Effect of CV on AFB_1_ Production

One hundred microliters of *A. flavus* mycelia solution (as described in Section 2.3.3) was added to 900-µL methanol, vortex-mixed for 2 min and centrifuged at 10,000× *g* r/min for 10 min. The supernatant was used as the AFB_1_ sample. The AFB_1_ samples were analyzed by an Agilent HPLC 1100 system equipped with a reverse-phase column Sycronis C18 (150 mm × 4.6 mm, 5 μm) at 35 °C and detected by a fluorescence detector (excitation and emission wavelengths 360 nm and 440 nm, respectively). The mobile phase conditions were as below: 40% methanol and 60% water, 0.8 mL/min flow rate and isocratic elution. The content of AFB_1_ was calculated by the standard curve y = 1.7841x − 6.024 (concentration range 6.25–400 μg/L).

#### 2.3.5. Scanning Electron Microscopy (SEM) Analysis

Mycelia were cultured as described in Section 2.3.3; 5-mm × 10-mm segments were filtered from mycelia and washed twice by 0.1-M phosphate-buffered saline (PBS, pH 7.2). The samples were fixed by 2.5% glutaraldehyde solution for a fixed 24 h and washed three times in 0.2-M phosphate-buffered saline (PBS, pH 7.4) for 15 min each time. The segments were dehydrated twice in graded ethanol in a series (15–100%) over a period of time for 20 min each time, then air-dried, fixed on the radio with a conductive adhesive, sprayed with gold for 30 s and, finally, observed by SEM. Samples were fixed by 2.5% glutaraldehyde solution for 24 h and washed three times in 0.2-M phosphate-buffered saline (PBS, pH 7.4) for 15 min each time. Sections were dehydrated twice (15–100%) in graded ethanol in series for 20 min each time, then air dried, fixed on radio with conductive gel, sprayed with gold for 30 s and, finally, observed by SEM.

### 2.4. Antifungal Mechanism of CV

#### 2.4.1. The Content of Ergosterol in Mycelia Plasma Membrane

The content of ergosterol in an *A. flavus* mycelia plasma membrane was determined as Tian et al. [13] described. *A. flavus* spores (100 μL, 2 × 10^8^ spores/mL) were inoculated into 50-mL liquid sabourand medium with different concentrations of CV (0, 50, 100 and 200 µg/mL) and cultured at 28 °C with a speed of 200 r/min for 2 days. Then, *A. flavus* mycelia were filtered and collected. Five milliliters of 25% alcoholic potassium hydroxide solution were added to each sample, vortex-mixed for 2 min and then incubated at 85 °C for 4 h. Two milliliters of sterile distilled water and 5-mL n-heptane were then added, followed by vortex mixing for 2 min. The n-heptane layer (upper layer) was collected and scanned in the range of 230–300 nm by UV spectrophotometry. The presence of ergosterol (at 282 nm) and 24(28) dehydroergosterol (at 230 and 282 nm) in mycelium led to a characteristic curve. The ergosterol amount was calculated as a percentage of the wet weight of the cells and was based on the absorbance and wet weight of the initial pellet. Samples without CV treatment were considered as the control group. The ergosterol amount was calculated by a formula as follows:

(1) (%) ergosterol =(A282/290)/net wet weight of mycelia−(%) 24 (28) dehydroergosterol

(2) %24 (28) dehydroergosterol = (A230/518)/net wet weight of mycelia

where *A*282 and *A*230 are the absorbance of the n-heptane layer in 282 nm and 230 nm, respectively. The E-values are 290 and 518 (in percentages per cm) determined for crystalline ergosterol and 24 (28) dehydroergosterol, respectively.

#### 2.4.2. Lipid Extraction from *A. flavus* Mycelia

Firstly, 50-mg dried *A. flavus* mycelia (as described in Section 2.3.3) was dissolved in 2-mL anhydrous alcohol. The lipid in *A. flavus* mycelia was extracted by ultrasonic extraction for 20 min. The mixture was centrifuged at 4500× *g* r/min for 10 min. Subsequently, the supernatant was pipetted into a Ostro 96-well plate, which was activated by 200-μL methanol. The lipid in the supernatant was adsorbed on the plate. Then, 2-mL chloroform/methanol/triethylamine (4.5/4.5/1, *v*/*v*/*v*) was added to elute the lipid. The collected lipids were dried by nitrogen blowing and dissolved in 200 µL of chloroform/methanol (2/1, *v*/*v*). Finally, the lipid in *A. flavus* mycelia was analyzed by the UPLC-HRMS system. Before the lipidomics analysis, the quality control samples (QC) were mixed equally from equal amounts of samples from each group (control group, 100 and 200 μg/mL). QC samples were used to balance the mass accuracy before injection and correct for peak intensity or retention time, and then, they were employed to evaluate the system stability during the experiment. Five QC samples were determined at the beginning of each run. One QC sample was determined after every 6 analyzed samples were measured. QC samples were used for filtering lipids during data processing, and in order to better show the relationship between the changes in the control and treatment groups, so quality control samples were not presented in the paper.

#### 2.4.3. Lipid Detection by UPLC-HRMS

The analysis of the mycelial lipids was achieved by the Dionex high-performance liquid chromatography system with tandem Thermo Orbitrap Fusion mass spectrometer (Thermo Fisher Scientific, Waltham, MA, USA). An ACQUITY UPLC BEH C18 column (100 mm × 2.1 mm, 1.7 µm) provided by Waters (Milford, MA, USA) was used for the separation of the target compounds. The temperature of the column was maintained at 40 °C, and the injection volume was 1 μL. Separations under a gradient elution at 0.2 mL/min based on acetonitrile/H_2_O (6:4, *v*/*v*) + 0.1% formic acid + 10-mmol/L ammonium formate (solvent A) and isopropanol/acetonitrile (9:1, *v*/*v*) + 0.1% formic acid + 10-mmol/L ammonium formate (solvent B) was as follows: 0–4 min 15% B, 5 min 48% B, 17 min 70% B, 20 min 82% B, 24 min 99% B and 24.2–30 min 15% B.

The following parameters were used for MS. The source parameters were as below: ion source type, ESI source; scan mode, positive (+)/negative (−) ion mode; spray voltage, 3500 v (+)/3000 v (−); ion transfer tube temperature, 320 °C and gas heater temperature, 320 °C. Both sheath gas and auxiliary gas were nitrogen, with the sheath gas flow 40 arb, auxiliary gas flow 5 arb and sweep gas flow 0 arb. The main parameters of the full scan were as follows: MS1, Orbitrap resolution, 240,000; scanning range, *m*/*z* 150–2000; RF lens (%), 60; AGC target, 1.0e6, MS2 and MS3, activation type, HCD; HCD collision energy (%), 35 ± 5; Orbitrap resolution, 30,000; AGC target, 5.0 × 10^4^ [26].

#### 2.4.4. Lipidomics Analysis of *A. flavus* Mycelia

The raw data obtained from UPLC-HRMS were collected by Xcalibur 4.0 software (Thermo Scientific, Waltham, MA, USA). Lipid identification used LipidSearch 4.0 software (Thermo Scientific) (Shanghai, China), which contained 18 lipid species and over 1,500,000 fragment ions to match the experiment data [27,28].

The adductions included +H^+^, +NH_4_^+^ and +2H^+^ for the positive mode and H^+^, +CHOO^-^ and −2H^+^ for the negative mode. Subsequently, the lipid molecules in the extracted data with missing values >50% were deleted. The missing values that were not eliminated were filled with 1/5 minimum values. After that, the data were processed with quality controls (QCs), and data with relative standard deviations (RSD) > 30% were eliminated, and the peak area was normalized. Preprocessed data were imported to Metaboanalyst 5.0 for the multivariate statistical analysis, including a principal component analysis (PCA) and orthogonal partial least squares discriminant analysis (OPLS-DA). Metaboanalyst 5.0, a web-based platform with rich compound databases and path libraries, has become widely used for comprehensive metabolomics data analysis, interpretation and multi-omics data integration of a wide range of species [29]. The significantly differential lipids between the control and CV treatment groups were selected using the protocol *p* < 0.05, fold change (FC) > 2.0 (upregulated) or 0.5 (downregulated) and variable importance in projection (VIP) > 1.0 [30]. The significantly different lipids (*p* < 0.05, VIP > 1) between the control group and 200-µg/mL CV-treated group were imported into a “pathway analysis” panel of Metaboanalyst 5.0 for the metabolic pathway analysis. The lipid metabolic pathway analysis was performed according to the Kyoto Encyclopedia of Genes and Genomes (KEGG) pathway database.

## 3. Results and Discussion

### 3.1. Antifungal Activity of CV

**Figure 1:**
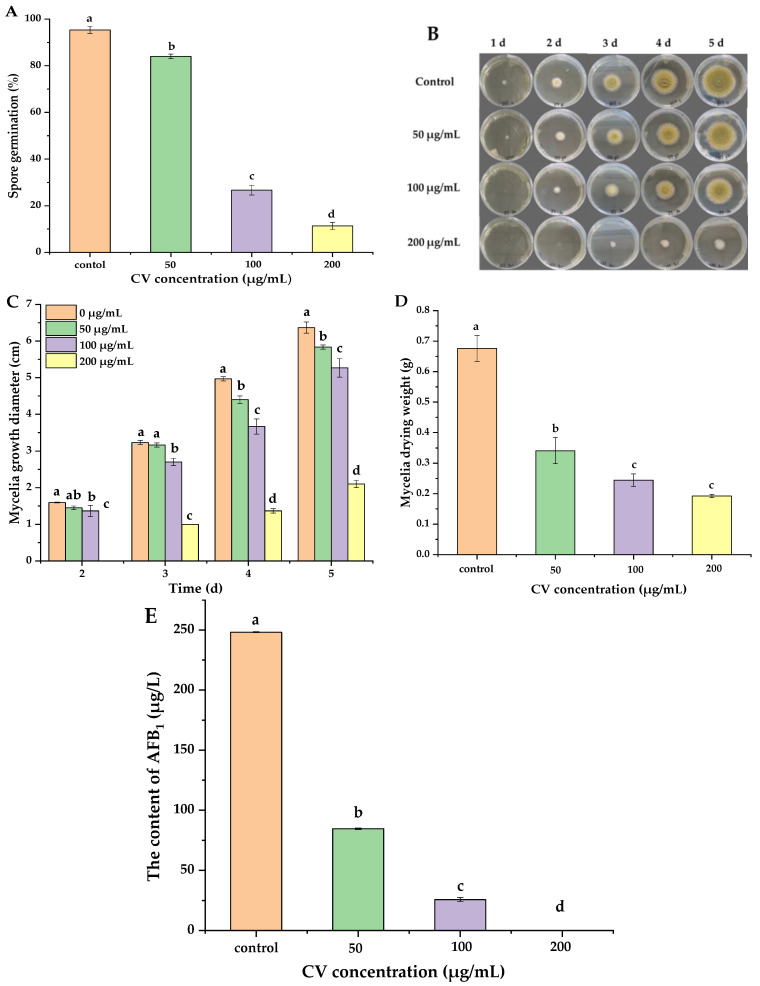
Antifungal activity of CV on *A. flavus*. (**A**) Spore germination, (**B**,**C**) colony growth diameter, (**D**) mycelia weight and (**E**) the content of AFB_1_. Different letters indicated significant differences (*p* < 0.05).

It can be seen from Figure 1A that spore germination of *A. flavus* was inhibited effectively by CV. Following 8 h, in the experimental group, with the increase of CV concentration, the percentage of *A. flavus* spore germination decreased. Specifically, the percent germination of 50-, 100- and 200-µg/mL CV-treated spores were 84.0% and 26.7% and 11.3%, respectively.

The inhibitory effect of CV at different concentrations on the mycelia growth of *A. flavus* is displayed in Figure 1B,C. In general, all tested concentrations of CV exhibited inhibition of mycelia growth, and the inhibition degree was concentration-dependent. After 5 days, compared to the mycelia diameter of the control (6.4 cm), a significant inhibition effect of CV on mycelia diameters at 50-µg/mL, 100-µg/mL and 200-µg/mL concentrations were found at 5.8 cm, 5.3 cm and 2.1 cm, respectively. As can be seen from the data, CV had a good inhibitory effect on the growth of *A. flavus*.

The antifungal effects of presenting different concentrations of CV on the dry mycelia weigh of *A. flavus* cultured for 5 days are displayed in Figure 1D. The concentration of CV was negatively correlated with the dry weight of mycelia. Compared to the control group (0.68 g), the dry weight of *A. flavus* mycelia treated with CV of 50 and 100 µg/mL decreased to 0.34 and 0.24 g, respectively. At the concentration of 200 µg/mL, the dry weight of mycelia was only 0.19 g.

The inhibitory effect of CV at different concentrations on the AFB_1_ is displayed in Figure 1E. CV had a significant inhibitory effect on AFB_1_ production. The AFB_1_ amount of the control group was 248.19 µg/l. After treated with 50-, 100- and 200-µg/mL CV, the amount of AFB_1_ were 84.58 µg/L, 25.78 µg/L and 0 µg/L, respectively.

### 3.2. CV Destroyed the Integrity of A. flavus Cell Membrane

The fungal cell membrane can absorb nutrients, exchange substances and energy with the surroundings and maintain cell viability [31]. Some essential oils may cause cell necrosis by damaging the cell membrane of *A. flavus* [8]. Since the growth and toxin production of *A. flavus* was significantly inhibited by CV, we wondered whether CV damaged the integrity of *A. flavus* cell membrane. Hence, we investigated the cell morphology using SEM.

**Figure 2:**
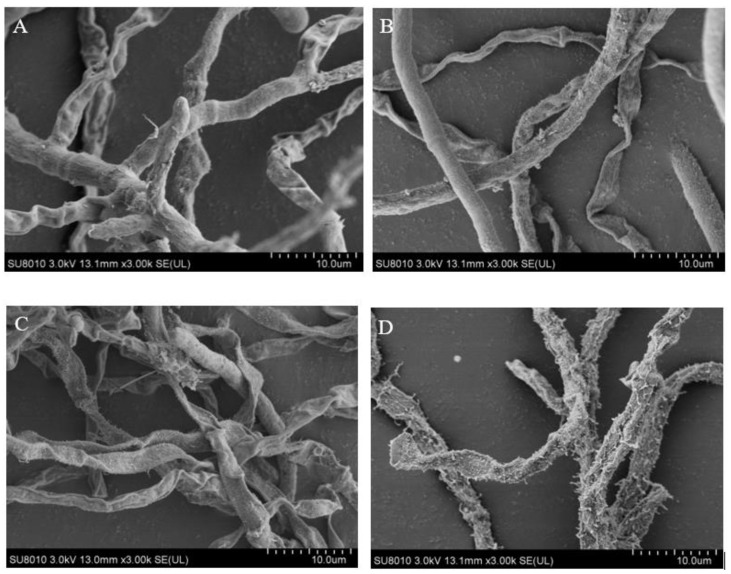
Effect of CV on mycelia structures from *A. flavus*. (**A**) Control group, (**B**) 50 μg/mL, (**C**) 100 μg/mL and (**D**) 200 μg/mL.

An intact morphology (the control) with striated linear, smooth hyphae is shown in the SEM image of *A. flavus* mycelia (Figure 2A). However, after being incubated with CV, a slightly shrunken appearance was observed. The deformation can be seen, including a rough and wrinkled surface, in the mycelia morphology following the 200-μg/mL treatment (Figure 2D). The reason for the rough and wrinkled surface of the *A. flavus* mycelia was the rupture of the membrane integrity and loss of the cytoplasmic contents [32].

### 3.3. Ergosterol Content of Mycelium

**Figure 3:**
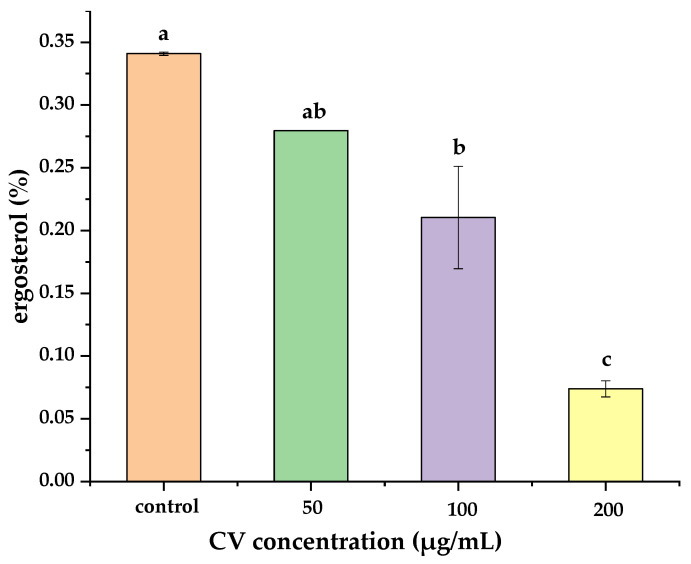
Ergosterol content of *A. flavus* mycelia. Different letters indicated significant differences (*p* < 0.05).

Ergosterol is a key component of the fungal cell membrane, which is an important substance to maintain membrane integrity and normal physiological function of fungal cells. It is also considered as a marker for estimating fungal biomass in different matrices [33]. The ergosterol content in the plasma membrane of *A. flavus* was determined, and the inhibition result of CV on the synthesis of ergosterol can be seen in Figure 3. The total ergosterol content was determined at 0-, 50-, 100- and 200-μg/mL concentrations of CV with values of 0.341 ± 0.001%, 0.230 ± 0.000%, 0.210 ± 0.041% and 0.074 ± 0.006%, respectively. Furthermore, the ergosterol contents of *A. flavus* mycelium after 100-μg/mL and 200-μg/mL CV treatment were significantly different from the control. The reduction of ergosterol content in *A. flavus* following treatment with different concentrations of CV suggested that the inhibitory effect of CV on the ergosterol content in the *A. flavus* cell membrane was shown in a dose-dependent manner.

### 3.4. UPLC-HRMS Lipid Metabolite Profiling of A. flavus

#### 3.4.1. Multivariate Analysis

Lipid extracts of 100-μg/mL and 200-μg/mL CV-treated *A. flavus* mycelia and the control group were analyzed in positive and negative ion modes using a UPLC-HRMS. A total of 369 lipid molecules in positive ionization mode and 368 lipid molecules in negative ionization mode were detected. The subclasses of the defined lipids of *A. flavus* could be classified into ceramides (Cer), monoglycosylceramide (CerG1), diglycosylceramide (CerG2), triglycosyl-ceramide (CerG3), ceramides phosphate (CerP), cardiolipin (CL), cyclic phosphatidic acid (cPA), dimethylphosphatidylethanolamine (dMePE), diglyceride (DG), digalactosyldiacylglycerol (DGDG), digalactosylmonoacylglycerol (DGMG), fatty acid (FA), monoglyceride (MG), monogalactosyldiacyiglycerol (MGDG), monogalactosyl monooctanoyl glycerol (MGMG), lysodimethylplosphatidylethanolamine (LdMePE), lysophosphatidic acid (LPA), lysophosphatidylcholines (LPC), lysophosphatidylethanol (LPEt), phosphatidylethanolamines (LPE), lysophosphatidylmethanol (LPMe), lyso-lysophosphatidylinositol (LPI), phosphatidylglycerols (LPG), lipopolysaccharide (LPS), phosphatidic acid (PA), platelet-activating factor (PAF), phosphatidylcholines (PC), phosphatidylethanolamine (PE), phosphatidylethanol (PEt), phosphatidylinositols (PI), phosphatidylinositrol (PIP, PIP2), phosphatidylglycerols (PG), phosphatidylmethanol (PMe), phosphatidylserine (PS), sphingomyelin (SM), sphingoshine (So), sphingoshine phosphate (SoP), sulfoquinovosyldiacylglycerol (SQDG), sulfoquinovosylmonoacylglycerol (SQMG), triglyceride (TG), (O-acyl)-1-hydroxy fatty acid (OAHFA) and sphingomyphlin (phytosphingosine) (phSM).

**Figure 4:**
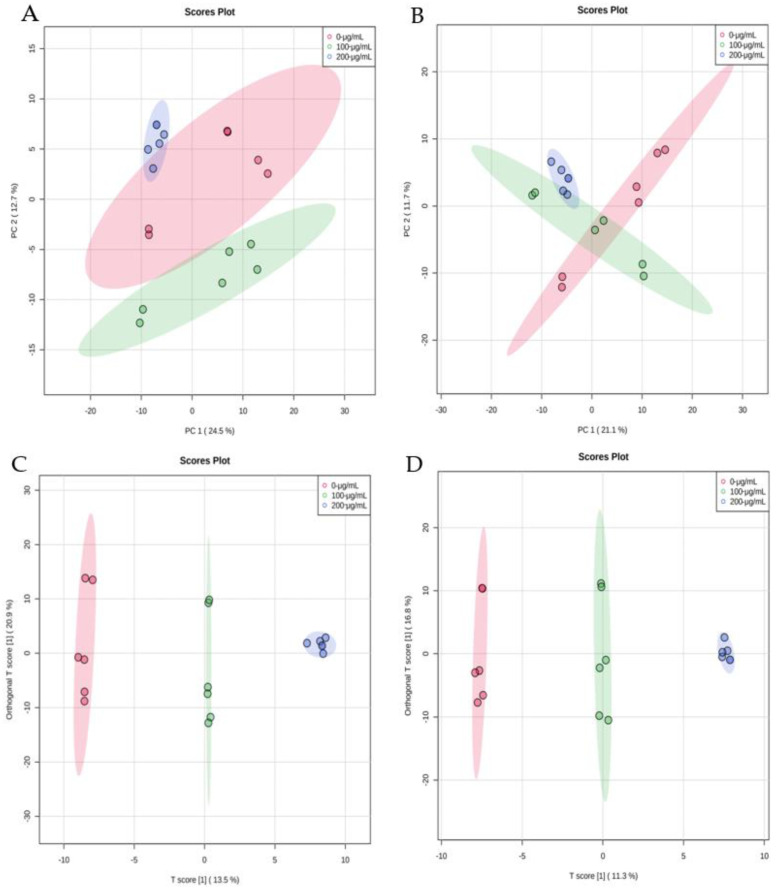
PCA score plots based on the UPLC-HRMS data of CV-treated groups and the control group in the positive mode (**A**) and in the negative mode (**B**), and OPLS-DA score plots of CV-treated groups and the control group in the positive mode (**C**) and in the negative mode (**D**).

As shown in Figure 4A,B, the explained variance by the first two principal components in the control group (red dots), 100 μg/mL (green dots) and 200 μg/mL group (blue dots) were 24.5% (PC1) and 12.7% (PC2) in the positive mode and 21.1% (PC1) and 11.7% (PC2) in the negative mode. Furthermore, the blurred boundary among the areas occupied groups can be seen in the negative mode. In order to obtain clearer separations, a supervised pattern recognition method, OPLS-DA, was used to discover significant discriminants among the samples. In the OPLS-DA score plots, the control, 100- and 200-μg/mL CV-treated groups were clearly distinguished in positive and negative modes. To ensure that the calculated models were reliable, 100 times the permutation was performed with the model parameters in positive mode (R^2^Y = 0.997, Q^2^ = 0.858) and negative mode (R^2^Y = 0.999, Q^2^ = 0.706), showing a more predictive performance and goodness-of-fit results (Figure 4C,D). Both positive and negative mode datasets presented clear segregation among the three groups, showing that the lipid metabolism of *A. flavus* was affected by CV significantly.

Based on the OPLS-DA models (Figure 4C,D), the 100-μg/mL CV-treated group was closer to the control group when compared to the 200-μg/mL CV-treated group, indicating that lipid metabolism was dependent on CV concentration. Variable important in projection (VIP) scores from the OPLS-DA models were calculated. Lipids with VIP > 1 and *p*-values < 0.05 were regarded as significantly different metabolites. A total of 88 differential lipid metabolites were selected according to their VIP and *p*-values, which are shown in Table 1. In addition, glycerolipids (in the positive ion mode) and glycerophospholipids (in the negative ion mode) were the main significant differential lipids between the control group and CV-treated groups.

#### 3.4.2. Univariate Statistical Analysis

To better understand the possible antifungal mechanism of CV to *A. flavus*, metabolic differences between the control and CV-treated groups (volcano plots) were investigated to recognize the lipids that showed significant differences (*p* < 0.05, FC > 2).

**Figure 5:**
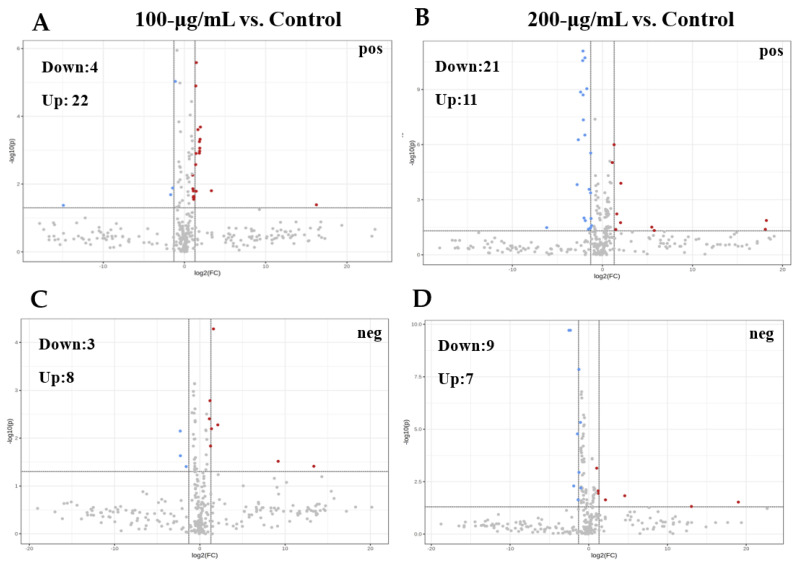
Volcano plot analysis of 100μg/mL CV-treated vs. the control in the positive (**A**) and negative (**C**) modes, and 200-μg/mL CV-treated vs. the control in the positive (**B**) and negative (**D**) mode.

Compared with the control group, four lipids downregulated while 22 lipids upregulated for the 100-μg/mL CV treated group (Figure 5A), and 21 lipids downregulated while 11 lipids upregulated for the 200-μg/mL CV-treated group in the positive ion mode (Figure 5B). The significantly differential lipids are supplied in Supplementary Materials Table S1. In addition, in the negative ion mode, three lipids were downregulated with eight lipids upregulated (100-μg/mL CV-treated, Figure 5C) and nine lipids downregulated with seven lipids upregulated (200-μg/mL CV-treated, Figure 5D) in comparison with the control. The significantly differential lipids were supplied in Supplementary Materials Table S2. Furthermore, TG (18:2/17:2/18:2), TG (18:3/18:2/18:3), TG (18:2/18:2/16:2), TG (18:2/18:2/18:3), TG (25:0/18:2/18:2), TG (15:0/18:2/18:3), TG (24:3/12:0/21:4), TG (16:0/16:1/21:4), dMePE (18:2/13:0) and PE (18:2/15:0) are significantly downregulated with increasing CV concentrations in Supplementary Materials Table S3.

#### 3.4.3. Hierarchical Clustering Analysis

**Figure 6:**
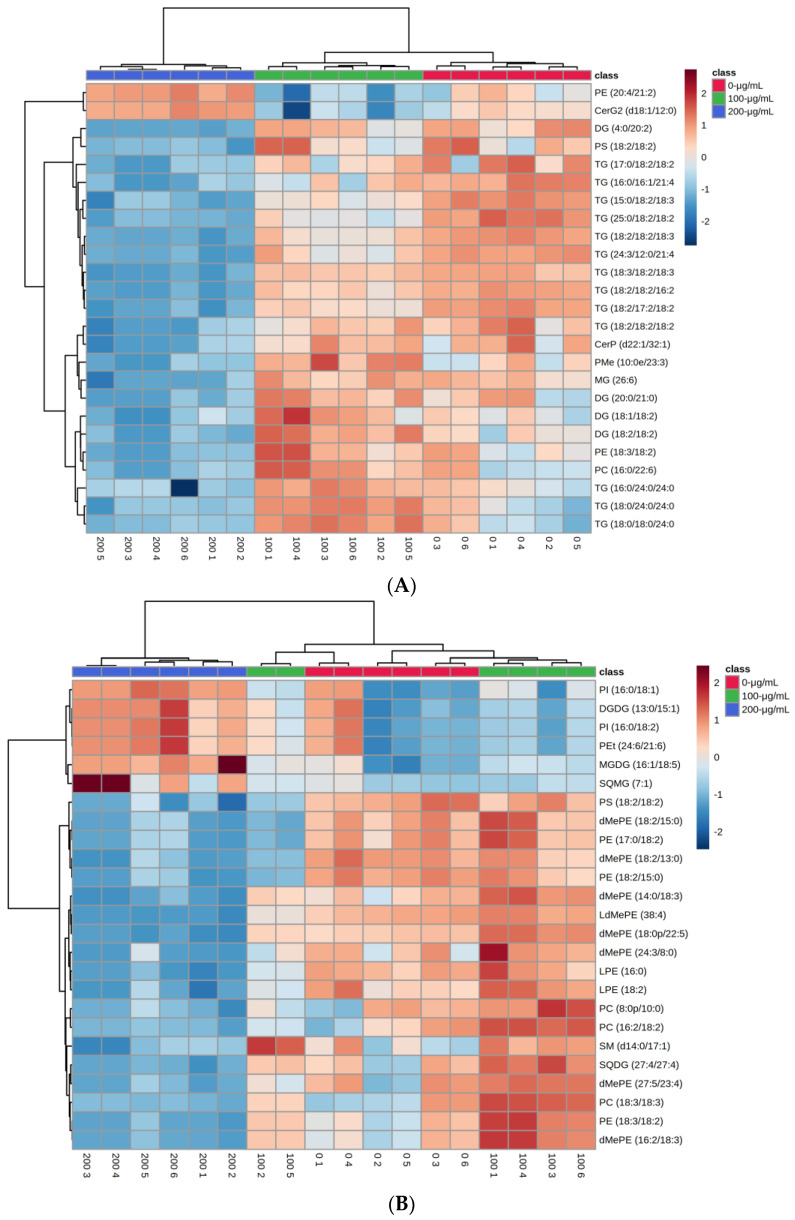
Hierarchical clustering heat map of the 25 top significant differential metabolites, with the degree of change marked with red (upregulated) and blue (downregulated) (**A**) in the positive mode and (**B**) in the negative mode.

To observe the discrimination trend in more detail and evaluate the rationality of differential metabolites, a hierarchical clustering heat map of up- and downregulated significantly differential lipids in *A. flavus* treated by CV were performed to show the overall trend of the 25 top significant ion features. From Figure 6A, we can find that most of the significant features expressed a downregulated trend after being 200-μg/mL CV-treated compared with the control group and 100-μg/mL CV-treated, principally for triglycerides. A study in yeast showed that TG was a novel lifespan-promoting factor, stored more TG in yeast cells and had a significantly extended lifespan than those cells depleted of TG [34]. Thus, treatment with 200-μg/mL CV may not be conducive to mycelial life extension. From Figure 6B, in the negative mode, specific lipid classes (e.g., PI, DGDG, MGDG and SQMG) were upregulated with 200-μg/mL CV treatment. PIs have physiological regulatory functions in membrane trafficking, membrane cytoskeletal interactions and ion channel and transporter activity [21], and the upregulation of PIs and glycolipids 200-μg/mL CV-treated may trigger the relevant signaling pathways.

#### 3.4.4. Analysis of Metabolic Pathways

The metabolic pathway analysis was of great significance in revealing the antifungal mechanism of CV and understanding the metabolic process alteration of *A. flavus* after CV treatment. The significantly different lipids (*p* < 0.05, VIP > 1) between the control group and 200-µg/mL CV-treated group were imported into the pathway analysis panel of Metaboanalyst 5.0 for the metabolic pathway analysis.

**Figure 7:**
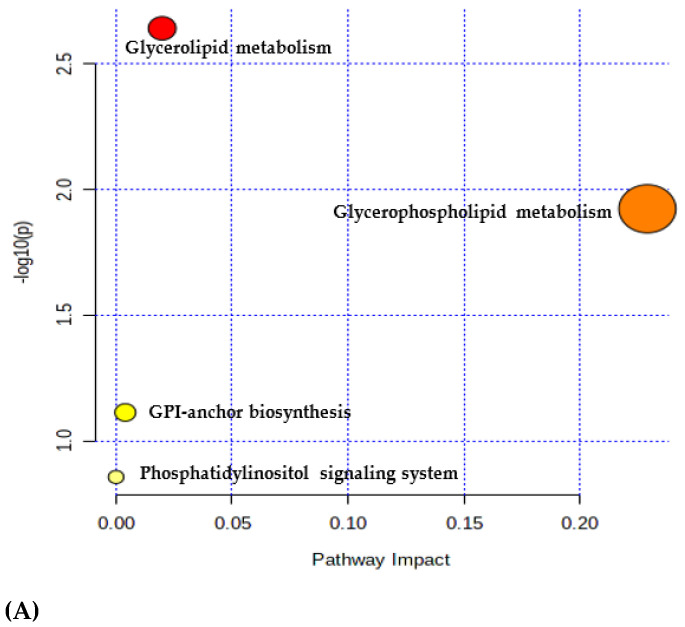

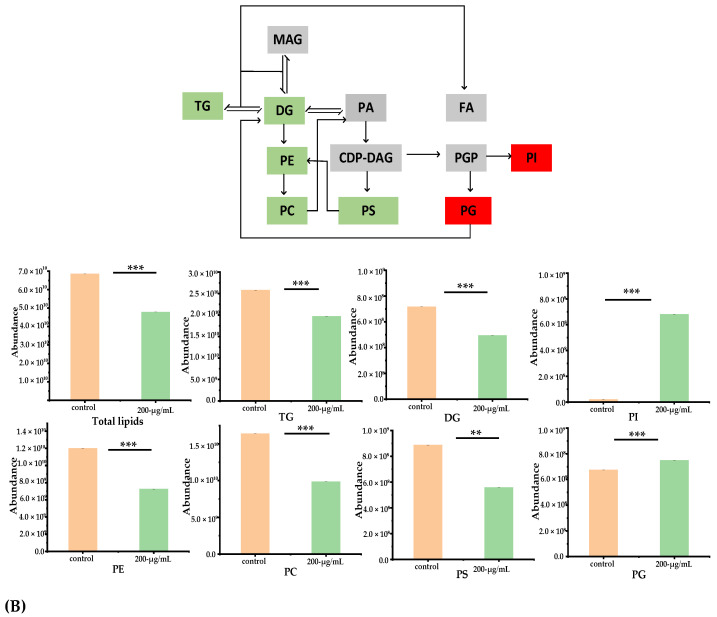
(**A**) Pathway analysis overview depicting altered metabolic pathways in *A. flavus* from the 200-μg/mL CV-treated group and the control. (**B**) Biosynthesis of glycerolipids and phospholipids [35] in *A. flavus* [36]. The red boxes showed upregulated lipids of mycelium treated with 200-μg/mL CV vs. the control, the green boxes indicated downregulated lipids and the gray boxes were lipids undetected or not changed significantly in the pathways. The glycerolipids included monoacylglycerol (MAG), DG and TG, whereas the phospholipids included PA, PC, PE, PS, PI and PG. CDP-DAG: CDP-diacylglycerol, PGP: proline-glycine-proline and FA: fatty acids. The bar groups indicated the abundance of lipids. Data were presented as the mean ± standard error of the mean (*n* = 6). ** and *** indicated *p* < 0.01 and 0.001, respectively.

As shown in Figure 7A and Table 2, four metabolic pathways of *A. flavus* were affected by CV at 200 μg/mL, including glycerophospholipid metabolism, glycosylphosphatidylinositol-anchor biosynthesis (GPI-B) and glycerolipid metabolism and phosphatidylinositol signaling. Among these pathways, only one distinctly altered pathway, glycerophospholipid metabolism, was filtered based on specific criteria (raw *p* < 0.05 and impact value > 0.1). The biosynthesis of glycerolipids and phospholipids in *A. flavus* is shown in Figure 7B. The glycerolipids (TG and DG) and phospholipids (PC, PE and PS) were significantly downregulated, whereas PI and PG were upregulated. The abundance of the lipid metabolites and total lipids in the selected pathways indicated that the CV treatment had a potent inhibition of the growth of *A. flavus*.

## 4. Discussion

CV is one of the potent monoterpenes that can be utilized to control fungal species [37]. In the present study, we determined the effectiveness of CV on inhibiting the growth of *A. flavus* both in the agar and liquid phases. In our study, spore germination, mycelia growth and aflatoxin production of *A. flavus* were significantly inhibited by CV. Similar results for the antifungal effect of CV were also found in *Fusarium oxysporum*, *Neocosmospora solani* and *Microdochium nivale*, and CV at a 0.2–0.4-mg/mL concentration also inhibited the spore germination in a dose-dependent manner [37]. In addition, CV also made the mycelia of *A. flavus* shrink and form a rough surface. Our results confirmed that CV was able to damage the cell membrane and reduced the ergosterol content of *A. flavus*. Cell disruption in the cell membrane and imbalance in cell permeability may lead to the loss or reduction of the ergosterol content [38]. Similar results were found for the ergosterol content of *A. flavus* treated with 0.6-μL/mL *Cuminum cyminum* L. seed essential oil [39]. Furthermore, Xu et al. found that nine differentially expressed genes involved in ergosterol synthesis were downregulated after exposure of *A. flavus* mycelia to 0.25 μL/mL of cuminaldehyde [8].

Lipids have long been believed to have a structural role in the biomembrane and a role in energy storage [40]. Therefore, in order to investigate the antifungal mechanism of CV from a new perspective, lipidomic technology based on UPLC-HRMS was used to detect and analyze the metabolites of *A. flavus* after CV treatment. Our data suggested that CV concentrations at 100 μg/mL or greater showed evident mycelia growth and AFB_1_ production inhibition compared to the control group. In order to explore lipid metabolism changes caused by different concentrations of CV, 100 and 200 μg/mL were chosen for further study. The inhibitory mechanism of CV against *A. flavus* was studied using a lipidomic metabolism analysis.

Most of the significant differential lipids of *A. flavus* mycelia were downregulated after 200-μg/mL CV treatment. Li et al. investigated the antifungal efficacy of paeonol on *A. flavus*; they found that the lipid content and glycero content of mycelia treated with paeonol at the minimum inhibitory concentration (MIC) was remarkably reduced by 46.21% and 64.68% [25]. Similar results of the total lipids content reduction were also found in α-phellandrene, nonanal-treated *P. cyclopium* [41] and Mentha piperita essential oil-treated *Fusarium sporotrichioides* [42]. The mechanism of action resulting in the reduction of the lipid content was that α-phellandrene oil and nonanal had the ability to penetrate the lipid structure of the cell and disrupt the integrity of the cell membrane [43]. *Mentha piperita* essential oil was capable of acting on the cell membrane structure and disrupting the cell membrane integrity. The result of reduction of the glycerol content was similar with pyrrolnitrin-treated *Neurospora crassa,* which could be the inhibition of respiration of *Neurospora crassa* by pyrrolizidine [44].

It was found that significant differential metabolites were involved in four metabolic pathways in the *A. flavus* mycelia. The glycerophospholipids metabolic pathway was a potential target metabolic pathway for CV intervention. Glycerophospholipids were key components of the cellular lipid bilayer, and their fatty acid compositions have important effects on membrane characteristics, such as membrane fluidity, transport triacylglycerols and cholesterol in the body and the formation of lipid rafts [43]. The glycerophospholipid bilayer was an integral cell membrane structure that played an important role in protecting cellular components from external influences and maintaining internal cellular biological functions without interference [37]. At the same time, glycerophospholipid metabolism was essential to the cell membrane dynamics [45]. Thus, the amounts of some kinds of glycerophospholipids induced by external factors may affect the overall cell metabolism. Moreover, the lipids associated with cell membrane homeostasis-related pathways, such as glycerophospholipid metabolism, were evidentially downregulated upon 200-μg/mL CV treatment.

The decrease of the abundance of most of the glycerolipids and phospholipids of *A. flavus* indicated the inhibition of the lipid metabolism by CV. Neutral lipids in the fungi primarily included TGs, which were primarily responsible for the storage of free fatty acids, sterols and DG [21]. The general pattern of neutral lipid turnover in many fungi manifests itself as a mechanism of resistance to chemical fungicides [46,47,48]. After treatment with 200-μg/mL CV, the abundance of neutral lipid (TG) was downregulated. Similar results were found for the decreased neutral lipids in *Aspergillus parasiticus spear* treated with propolis [49]. Phospholipids, which are the most abundant lipids in cells, account for 40–60% of the total lipids in eukaryotic cells [50]. After treatment with 200-μg/mL CV, the abundance of PC, PE and PS was significantly downregulated, whereas PI and PG were upregulated, indicating a blocking in the pathway of other phospholipids fractions from PI and PG.

## 5. Conclusions

In this study, we discovered that CV had a high potential to reduce spore germination, mycelia growth, aflatoxin production and the ergosterol content of *A. flavus*. In addition, CV also made the mycelia of *A. flavus* shrink and form a rough surface. Most of the significant differential lipids of *A. flavus* mycelia were downregulated after 200-μg/mL CV treatment. In addition, the analysis of different lipid metabolic pathways between the control and 200-μg/mL CV-treated groups showed a significant enrichment of lipids in the glycerophospholipid metabolic pathway. This work deeply elucidated the lipidomics changes contributed to the prevention of *A. flavus* growth and the promotion of cell apoptosis, which is promising for providing novel molecular targets for the prevention of *A. flavus* contamination. These results provide a new perspective for the antifungal mechanisms of CV and promote its application as an antifungal agent.

## Figures and Tables

**Table 1:** Significantly altered lipid metabolites among the control and the CV-treated groups by the ANOVA test analysis with Fisher’s post hot analysis and OPLS-DA analysis.

No.	Extract Mass	Compound	*p* Value	VIP
1	(M + H)^+^	PC (16:2/18:2)	2.07 × 10^−3^	1.039
2	(M+ H)^+^	PE (20:4/21:2)	2.08 × 10^−5^	1.056
3	(M + NH_4_)^+^	TG (18:1/24:0/24:0)	2.50 × 10^−3^	1.090
4	(M + H)^+^	PC (16:0/22:6)	3.47 × 10^−5^	1.113
5	(M + H)^+^	PS (30:0/18:3)	8.21 × 10^−4^	1.125
6	(M + NH_4_)^+^	DG (10:0e/22:6)	6.62 × 10^−6^	1.158
7	(M + NH_4_)^+^	DG (18:1/18:2)	5.94 × 10^−5^	1.207
8	(M + NH_4_)^+^	TG (16:0/24:0/24:0)	8.55 × 10^−5^	1.284
9	(M + H)^+^	PE (18:2/18:1)	5.00 × 10^−3^	1.292
10	(M + H)^+^	PMe (10:0e/23:3)	1.58 × 10^−6^	1.312
11	(M + NH_4_)^+^	DG (18:2/18:2)	9.25 × 10^−8^	1.327
12	(M + NH_4_)^+^	DG (20:0/21:0)	8.58 × 10^−6^	1.544
13	(M + H)^+^	PE (18:3/18:2)	1.33 × 10^−5^	1.552
14	(M + H)^+^	DG (9:0/21:5)	1.63 × 10^−5^	1.586
15	(M + NH_4_)^+^	TG (16:0/18:2/24:0)	1.91 × 10^−3^	1.711
16	(M + NH_4_)^+^	TG (17:0/18:2/24:0)	6.75 × 10^−3^	1.799
17	(M + NH_4_)^+^	LdMePE (20:1p)	2.05 × 10^−4^	1.800
18	(M + NH_4_)^+^	TG (18:0/18:1/18:1)	3.95 × 10^−3^	1.840
19	(M + NH_4_)^+^	TG (18:1/18:2/18:2)	4.26 × 10^−3^	1.842
20	(M + NH_4_)^+^	TG (18:2/18:2/23:0)	4.26 × 10^−3^	1.891
21	(M + H)^+^	PS (18:2/18:2)	6.80 × 10^−4^	1.902
22	(M + NH_4_)^+^	TG (18:1/18:2/24:0)	1.11 × 10^−3^	1.906
23	(M + H)^+^	CerP (d22:1/32:1)	2.29 × 10^−5^	1.989
24	(M + NH_4_)^+^	TG (16:0/18:1/24:0)	8.24 × 10^−4^	1.991
25	(M + NH_4_)^+^	TG (18:0/17:0/18:2)	2.17 × 10^−4^	1.999
26	(M + H)^+^	MG (26:6)	2.93 × 10^−8^	2.052
27	(M + NH_4_)^+^	TG (16:0/17:0/18:1)	5.77 × 10^−4^	2.052
28	(M + NH_4_)^+^	TG (17:0/18:2/18:2)	2.41 × 10^−4^	2.121
29	(M + NH_4_)^+^	TG (18:0/18:1/24:0)	1.23 × 10^−6^	2.155
30	(M + H)^+^	PE (18:2/15:0)	3.34 × 10^−4^	2.169
31	(M + NH_4_)^+^	TG (26:0/18:0/18:1)	3.03 × 10^−5^	2.201
32	(M + NH_4_)^+^	TG (18:2/18:2/18:2)	3.98 × 10^−6^	2.217
33	(M + H)^+^	DG (4:0/20:2)	1.28 × 10^−7^	2.309
34	(M + NH_4_)^+^	TG (18:3/18:2/18:3)	2.06 × 10^−13^	2.396
35	(M + NH_4_)^+^	TG (18:0/18:0/18:1)	1.44 × 10^−5^	2.406
36	(M + NH_4_)^+^	TG (16:0/16:1/21:4)	1.99 × 10^−6^	2.464
37	(M + NH_4_)^+^	TG (18:2/18:2/16:2)	2.17 × 10^−13^	2.528
38	(M + NH_4_)^+^	TG (24:3/12:0/21:4)	1.65 × 10^−9^	2.545
39	(M + NH_4_)^+^	TG (18:0/17:0/18:1)	2.56 × 10^−8^	2.580
40	(M + NH_4_)^+^	TG (18:2/17:2/18:2)	3.32 × 10^−12^	2.591
41	(M + NH_4_)^+^	TG (18:2/18:2/18:3)	6.14 × 10^−12^	2.593
42	(M + NH_4_)^+^	TG (15:0/18:2/18:3)	3.01 × 10^−10^	2.618
43	(M + NH_4_)^+^	TG (25:0/18:2/18:2)	9.93 × 10^−11^	2.648
44	(M − H)^−^	CerG1 (d17:0/15:0)	1.52 × 10^−3^	1.846
45	(M − H)^−^	DGDG (13:0/15:1)	6.76 × 10^−3^	1.807
46	(M − H)^−^	dMePE (14:0/18:3)	1.44 × 10^−7^	1.998
47	(M − H)^−^	dMePE (15:0/16:1)	5.01 × 10^−5^	2.298
48	(M − H)^−^	dMePE (16:2/18:3)	1.32 × 10^−6^	1.448
49	(M − H)^−^	dMePE (18:0p/22:5)	1.24 × 10^−9^	2.185
50	(M − H)^−^	dMePE (18:2/13:0)	4.55 × 10^−5^	2.527
51	(M − H)^−^	dMePE (18:2/15:0)	1.80 × 10^−3^	2.147
52	(M − H)^−^	dMePE (18:2/16:1)	3.16 × 10^−3^	1.296
53	(M − H)^−^	dMePE (24:3/8:0)	3.62 × 10^−4^	1.879
54	(M − H)^−^	dMePE (25:3/8:0)	1.35 × 10^−3^	2.266
55	(M − H)^−^	dMePE (27:5/23:4)	6.32 × 10^−4^	1.637
56	(M − H)^−^	LdMePE (38:4)	5.74 × 10^−9^	2.504
57	(M − H)^−^	LPE (16:0)	1.76 × 10^−6^	2.445
58	(M − H)^−^	LPE (18:2)	2.46 × 10^−5^	2.148
59	(M − H)^−^	MGDG (12:0/18:2)	5.26 × 10^−3^	1.991
60	(M + HCOO)^−^	MGDG (12:0/23:1)	5.76 × 10^−3^	1.733
61	(M − H)^−^	MGDG (16:1/18:5)	1.07 × 10^−4^	2.429
62	(M − H)^−^	MGDG (16:1/25:9)	6.67 × 10^−3^	1.692
63	(M − H)^−^	PA (16:0/18:1)	6.60 × 10^−3^	2.003
64	(M − H)^−^	PA (18:2/18:2)	4.41 × 10^−3^	1.819
65	(M − H)^−^	PA (27:6/20:5)	6.51 × 10^−3^	1.692
66	(M + HCOO)^−^	PC (16:2/18:2)	1.14 × 10^−3^	1.389
67	(M + HCOO)^−^	PC (18:1/18:1)	4.01 × 10^−3^	1.349
68	(M + HCOO)^−^	PC (18:3/18:3)	6.06 × 10^−5^	1.080
69	(M + HCOO)^−^	PC (8:0p/10:0)	1.57 × 10^−3^	1.491
70	(M − H)^−^	PE (15:0/18:1)	8.70 × 10^−4^	1.834
71	(M − H)^−^	PE (17:0/18:2)	2.09 × 10^−3^	2.126
72	(M − H)^−^	PE (18:2/15:0)	8.34 × 10^−5^	2.490
73	(M − H)^−^	PE (18:2/18:1)	3.34 × 10^−3^	1.291
74	(M − H)^−^	PE (18:3/18:2)	9.03 × 10^−7^	1.495
75	(M − H)^−^	PEt (16:0/16:1)	5.94 × 10^−3^	2.018
76	(M − H)^−^	PEt (24:6/21:6)	5.53 × 10^−3^	1.838
77	(M − H)^−^	PEt (26:4/8:0)	4.74 × 10^−3^	1.556
78	(M − H)^−^	PI (16:0/18:1)	3.30 × 10^−3^	1.993
79	(M − H)^−^	PI (16:0/18:2)	4.81 × 10^−3^	1.818
80	(M − H)^−^	PI (18:2/18:2)	6.06 × 10^−3^	1.339
81	(M − H)^−^	PMe (16:0/17:1)	6.89 × 10^−3^	2.002
82	(M − H)^−^	PMe (17:1/18:2)	5.26 × 10^−3^	1.991
83	(M − H)^−^	PMe (18:2/17:2)	4.13 × 10^−3^	1.318
84	(M − H)^−^	PMe (4:0/8:0)	4.88 × 10^−6^	1.341
85	(M − H)^−^	PS (18:2/18:2)	1.10 × 10^−4^	2.442
86	(M+HCOO)^−^	SM (d14:0/17:1)	1.28 × 10^−5^	1.114
87	(M − H)^−^	SQDG (27:4/27:4)	4.22 × 10^−6^	1.561
88	(M+HCOO)^−^	SQMG (7:1)	4.31 × 10^−3^	1.921

**Table 2:** Results from the ingenuity pathway analysis with Metaboanalyst in the 200-μg/mL group vs. control group.

Pathway Name	Total ^1^	Hits ^2^	Raw *p*	−log10 (*p*)	Holm Adjust *p* ^3^	FDR ^4^	Impact ^5^
Glycerophospholipid metabolism	32	2	0.012	1.924	0.86	0.43	0.229
Glycerolipid metabolism	14	2	0.002	2.642	0.17	0.17	0.020
Glycosylphosphatidylinositol (GPI)-anchor biosynthesis	14	1	0.077	1.113	1	1	0.004
Phosphatidylinositol signaling system	26	1	0.139	0.856	1	1	0

Total ^1^ means the number of compounds in the pathway. Hits ^2^ is the actually matched number from the user uploaded lipids. Holm adjust *p* ^3^ is the *p*-value adjusted by the Holm–Bonferroni method. FDR ^4^ is the *p*-value adjusted using the false discovery rate. Impact ^5^ is the pathway impact value calculated from the pathway topology analysis.

## Data Availability

The data supporting the findings of this study are included within the article.

## Supplementary Materials

The following are available online at https://www.mdpi.com/article/10.3390/foods11010093/s1, Table S1: Significially differential lipids among 100 μg/mL vs. control group, Table S2: Significially differential lipids among 200 μg/mL vs. control group, Table S3: Significantly down-regulated lipids in a CV dose-dependent manner.

## References

[1] Mateo E.M., Gómez J., Gimeno-Adelantado J.V., Romera D., Jiménez M. (2017). Assessment of azole fungicides as a tool to control growth of *Aspergillus flavus* and aflatoxin B_1_ and B_2_ production in maize. Food Addit. Contam. Part A.

[2] Taniwaki M.H., Pitt J.I., Magan N. (2018). Aspergillus species and mycotoxins: Occurrence and importance in major food commodities. Curr. Opin. Food Sci..

[3] Kos J., Hajnal E.J., Malachová A., Steiner D., Sulyok M. (2019). Mycotoxins in maize harvested in Republic of Serbia in the period 2012-2015. Part 1: Regulated mycotoxins and its derivatives. Food Chem..

[4] Brr A., Mis B. (2019). Aflatoxin B_1_: A review on metabolism, toxicity, occurrence in food, occupational exposure, and detoxification methods. Food Chem. Toxicol..

[5] IARC (2012). Aflatoxins, IARC Monographs on the Evaluation of Carcinogenic Risks on Humans.

[6] Avanço G.B., Ferreira F.D., Bomfim N.S., Peralta R.M., Brugnari T., Mallmann C.A., de Abreu Filho B.A., Mikcha J.M., Machinski M. (2016). *Curcuma longa* L. essential oil composition, antioxidant effect, and effect on *Fusarium verticillioides* and fumonisin production. Food Control..

[7] Tian J., Ban X., Zeng H., Huang B., He J., Wang Y. (2011). In vitro and in vivo activity of essential oil from dill (*Anethum graveolens* L.) against fungal spoilage of cherry tomatoes. Food Control..

[8] Xu D., Wei M.Q., Peng S.R., Mo H.Z., Hu L., Yao L.S., Hu L.B. (2021). Cuminaldehyde in cumin essential oils prevents the growth and aflatoxin B_1_ biosynthesis of *Aspergillus flavus* in peanuts. Food Control..

[9] Prakash B., Media A., Mishra P.K., Dubey N.K. (2015). Plant essential oils as food preservatives to control moulds, mycotoxin contamination and oxidative deterioration of agri-food commodities—Potentials and challenges. Food Control..

[10] Ascencion L.C., Liang W.J., Yen T.B. (2015). Control of *Rhizoctonia solani* damping-off disease after soil amendment with dry tissues of Brassica results from increase in Actinomycetes population. Biol. Control..

[11] Ardakani S., Heydari A., Khorasani N., Arjmandi R. (2010). Development of new bioformulations of *Pseudomonas fluorescens* and evaluation of these products against damping-off of cotton seedlings. J. Plant Pathol..

[12] Hu Z.Y., Yuan K., Zhou Q., Lu C., Du L.H., Liu F. (2021). Mechanism of antifungal activity of Perilla frutescens essential oil against *Aspergillus flavus* by transcriptomic analysis. Food Control..

[13] Tian J., Ban X.Q., Zeng H., He J.S., Chen Y.X., Wang Y.W. (2012). The Mechanism of Antifungal Action of Essential Oil from Dill (*Anethum graveolens* L.) on *Aspergillus flavus*. PLoS ONE.

[14] Pinto L., Bonifacio M.A., De Giglio E., Cometa S., Logrieco A.F., Baruzzi F. (2020). Unravelling the Antifungal Effect of Red Thyme Oil (*Thymus vulgaris* L.) Compounds in Vapor Phase. Molecules.

[15] Kintzios S.E. (2002). Oregano: The Genera Origanum and Lippia.

[16] Tang X.R., Chen S.L., Wang L. (2011). Purification and identification of carvacrol from the root of Stellera chamaejasme and research on its insecticidal activity. Nat. Prod. Res..

[17] Yin H.B., Chen C.H., Kollanoor-Johny A., Darre M.J., Venkitanarayanan K. (2015). Controlling *Aspergillus flavus* and Aspergillus parasiticus growth and aflatoxin production in poultry feed using carvacrol and trans-cinnamaldehyde. Poultry Sci..

[18] Sharifi-Rad M., Varoni E.M., Iriti M., Martorell M., Setzer W.N., Contreras M.D., Salehi B., Soltani-Nejad A., Rajabi S., Tajbakhsh M. (2018). Carvacrol and human health: A comprehensive review. Phytother. Res..

[19] Lam S.M., Wang Z.H., Li B.W., Shui G.H. (2020). High-coverage lipidomics for functional lipid and pathway analyses. Anal. Chim. Acta..

[20] Züllig T., Kfeler H.C. (2020). High resolution mass spectrometry in lipidomics. Mass Spectrom. Rev..

[21] Pan J., Hu C.T., Yu J.H. (2018). Lipid biosynthesis as an antifungal target. J. Fungi.

[22] Gharwalová L., Kuliová M., Vasyliuk A., Mareová H., Kolouchová I. (2021). Sphingolipids of plant pathogenic fungi. Plant Protect. Sci..

[23] Helal G.A., Sarhan M.M., Shahla A.N.K.A. (2006). Effects of *Cymbopogon citratus* L. essential oil on the growth, lipid content and morphogenesis of Aspergillus niger ML2-strain. J. Basic Microb..

[24] Li Q., Zhao Y., Zhu X.M., Xie Y.L. (2021). Antifungal efficacy of paeonol on *Aspergillus flavus* and its mode of action on cell walls and cell membranes. LWT-Food Sci. Technol..

[25] Ferreira F.D., Kemmelmeier C., Arroteia C.C., Costa C., Mallmann C.A., Janeiro V., Ferreira F.M.D., Mossini S.A.G., Silva E.L., Machhinski M. (2013). Inhibitory effect of the essential oil of Curcuma longa L. and curcumin on aflatoxin production by *Aspergillus flavus* Link. Food Chem..

[26] Yzabc D., Xzabc D., Li T.A. (2021). Identification of glycerophospholipids using self-built recognition software based on positive and negative ion high-resolution mass spectrometric fragmentation experiments. Talanta.

[27] Chen H.P., Gao G.W., Liu P.X., Pan M.L., Chai Y.F., Liu X., Lu C.Y. (2018). Development and validation of an ultra performance liquid chromatography Q-Exactive Orbitrap mass spectrometry for the determination of fipronil and its metabolites in tea and chrysanthemum. Food Chem..

[28] Taguchi R., Ishikawa M. (2010). Precise and global identification of phospholipid molecular species by an Orbitrap mass spec-trometer and automated search engine Lipid Search. J. Chromatogr. A.

[29] Pang Z.Q., Chong J., Zhou G.Y., Morais D., Chang L., Barrete M., Gauthier C., Jacques P.E., Li S.Z., Xia J.G. (2021). MetaboAnalyst 5.0: Narrowing the gap between raw spectra and functional insights. Nucleic Acids Res..

[30] Jia W., Dong X.Y., Shi L., Chu X.G. (2020). Discrimination of milk from different animal species by foodomics approach based on high-resolution mass spectrometry. J. Agric. Food Chem..

[31] Sant D.G., Tupe S.G., Ramana C.V., Deshpande M.V. (2016). Fungal cell membrane-promising drug target for antifungal therapy. J. Appl. Microbiol..

[32] Sangmanee P., Hongpattarakere T. (2014). Inhibitory of multiple antifungal components produced by Lactobacillus plantarum K35 on growth, aflatoxin production and ultrastructure alterations of Aspergillus flavus and Aspergillus parasiticus. Food Control..

[33] Hu Y.C., Zhang J.M., Kong W.J., Zhao G., Yang M.H. (2017). Mechanisms of antifungal and anti-aflatoxigenic properties of essential oil derived from turmeric (*Curcuma longa* L.) on *Aspergillus flavus*. Food Chem..

[34] Handee W., Li X., Hall K.W., Deng X., Li P., Benning C., Williams B.L., Kuo M.H. (2016). An Energy-Independent Pro-longevity Function of Triacylglycerol in Yeast. PLoS Genetics.

[35] Vinayavekhin N., Kongchai W., Piapukiew J., Chavasiri W. (2020). *Aspergillus niger* upregulated glycerolipid metabolism and ethanol utilization pathway under ethanol stress. MicrobiologyOpen.

[36] Kanehisa M., Goto S. (2000). KEGG: Kyoto encyclopedia of genes and genomes. Nucleic Acids Res..

[37] Saghrouchni H., El Barnossi A., Salamatullah A.M., Bourhia M., Alzahrani A., Alkaltham M.S., Alyahya H.K., Tahiri N.E., Imtara H., Var I. (2021). Carvacrol: A promising environmentally friendly agent to fight seeds damping-off diseases induced by fungal species. Agronmy.

[38] Cai R., Hu M.M., Zhang Y.J., Niu C., Yue T.L., Yuan Y.H., Wang Z.L. (2019). Antifungal activity and mechanism of citral, limonene and eugenol against Zygosaccharomyces rouxii. LWT-Food Sci. Technol..

[39] Kedia A., Prakash B., Mishra P.K., Dubey N.K. (2014). Antifungal and antiaflatoxigenic properties of Cuminum cyminum (L.) seed essential oil and its efficacy as a preservative in stored commodities. Int. J. Food Microbiol..

[40] Van der Meer-Janssen Y.P.M., Van Galen J., Batenburg J.J., Helms J.B. (2010). Lipids in host-pathogen interactions: Pathogens exploit the complexity of the host cell lipidome. Prog. Lipid Res..

[41] Zhang J.H., Sun H.L., Chen S.Y., Zeng L., Wang T.T. (2017). Anti-fungal activity, mechanism studies on α-Phellandrene and Nonanal against Penicillium cyclopium. Bot. Stud..

[42] Rachitha P., Krupashree K., Jayashree G.V., Gopalan N., Khanum F. (2017). Growth inhibition and morphological alteration of Fusarium sporotrichioides by mentha piperita essential oil. Phcog. Res..

[43] Carrizo D., Chevallier O.P., Woodside J.V., Brennan S.F., Cantwell M.M., Cuskelly G., Elliott C.T. (2016). Untargeted metabolomic analysis of human serum samples associated with exposure levels of Persistent organic pollutants indicate important perturbations in Sphingolipids and Glycerophospholipids levels. Chemosphere.

[44] Okada A., Banno S., Ichiishi A., Kimura M., Yamaguchi I., Fujimura M. (2005). Pyrrolnitrin interferes with osmotic signal trans-duction in Neurospora crassa. J. Pestic. Sci..

[45] Rodriguez-Cuenca S., Pellegrinelli V., Campbell M., Oresic M., Vidal-Puig A. (2017). Sphingolipids and glycerophospholipids—The “ying and yang” of lipotoxicity in metabolic diseases. Prog. Lipid Res..

[46] Khashaba H.E. (1995). Physiological studies on some biologically acitive fungi isolated from the soil of the United Arab Emirates. Ph.D. Thesis.

[47] El-Mougith A.A. (1999). Effect of benomyl on the growth and lipid composition of Trichoderma koningii. Folia Microbiol..

[48] Fakas S.S., Papanikolaou M., Galiotou-Panayotou M.K., Aggelis G. (2006). Lipids of cunninghamella echinulate with emphasis to γ-linolenic acid distribution among lipid classes. Appl. Microbiol. Biotechnol..

[49] Hashem A., Abd-allah E.F., Alwathnani A.H. (2012). Effect of propolis on growth, aflatoxins production and lipid metabolism in *Aspergillus parasiticus spear*. Pak. J. Bot..

[50] Van M.G., Voelker D.R., Feigenson G.W. (2008). Membrane lipids: Where they are and how they behave. Nat. Rev. Mol. Cell Biol..

