# Supplementary material for: UHPLC-HRMS-Based Untargeted Lipidomics Reveal Mechanism of Antifungal Activity of Carvacrol against Aspergillus flavus
Source: Foods. 2021 Dec 30;11(1):93. doi: 10.3390/foods11010093 (PMC8750229; doi:10.3390/foods11010093)
Supplement: Supplementary file 1 [file foods-11-00093-s001.zip › foods-1501179-supplementary.pdf]

Supplementary Table S1. Significially differential lipids among 100 μg/mL vs control group.

Extract mass	Lipid	log2(FC)	Regulated
$(M+H)^+$	CerG1(d20:2/18:1)	-14.906	down
$(M+NH_4)^+$	TG(18:2/18:2/23:0)	-1.6927	down
$(M+NH_4)^+$	PG(18:0p/22:5)	-1.4545	down
$(M+NH_4)^+$	TG(25:0/18:2/18:2)	-1.1225	down
$(M+NH_4)^+$	TG(16:0/16:0/16:0)	1.0045	up
$(M+H)^+$	TG(4:0/13:0/14:1)	1.0379	up
$(M+H)^+$	PC(18:3/16:0)	1.0953	up
$(M+H)^+$	PC(18:3/18:3)	1.0976	up
$(M+H)^+$	CerG2(d13:0/15:1)	1.1045	up
$(M+H)^+$	CerG2(d13:0/14:0)	1.1369	up
$(M+H)^+$	PE(30:5/8:0)	1.1698	up
$(M+H)^+$	PC(15:1/20:4)	1.171	up
$(M+NH_4)^+$	TG(16:0/24:0/24:0)	1.382	up
$(M+H)^+$	DG(9:0/21:5)	1.3958	up
$(M+NH_4)^+$	TG(18:0/24:0/24:0)	1.4248	up
$(M+H)^+$	PC(16:0/22:6)	1.4425	up
$(M+NH_4)^+$	DG(10:0e/22:6)	1.4598	up
$(M+NH_4)^+$	TG(18:0/16:0/16:0)	1.658	up
$(M+NH_4)^+$	TG(18:0/18:0/24:0)	1.8312	up
$(M+NH_4)^+$	TG(18:0/16:0/22:0)	1.8448	up
$(M+NH_4)^+$	TG(26:0/18:0/18:0)	1.8652	up
$(M+NH_4)^+$	TG(18:0/16:0/24:0)	1.883	up
$(M+NH_4)^+$	TG(18:0/18:0/18:0)	1.9239	up
$(M+NH_4)^+$	TG(25:0/18:0/18:0)	1.9631	up
$(M+H)^+$	CerG1(d18:1/15:1)	3.3145	up
$(M+NH_4)^+$	TG(20:0p/19:5/23:2)	16.241	up
$(M-H)^-$	PMe(8:0/14:0)	-2.2992	down
$(M-H)^-$	SQDG(10:2/18:5)	-2.2834	down
$(M-H)^-$	cPA(11:0)	-1.6026	down
$(M-H)^-$	MGDG(15:2/15:2)	1.1184	up
$(M+HCOO)^-$	PC(18:3/16:0)	1.1883	up
$(M+HCOO)^-$	PC(18:3/18:3)	1.2553	up
$(M-H)^-$	CerG1(d17:0/15:0)	1.3625	up
$(M-H)^-$	PMe(4:0/8:0)	1.5965	up
$(M+HCOO)^-$	SQMG(7:2)	2.1032	up
$(M-H)^-$	dMePE(20:1/22:4)	9.1868	up
$(M-H)^-$	CerG2(d14:0/18:0)	13.368	up

Supplementary Table S2. Significially differential lipids among 200 μg/mL vs control group.

Extract mass	Lipid	log2(FC)	Regulated
$(M+H)^+$	dMePE(6:0/13:0)	-6.1866	down
$(M+NH_4)^+$	TG(18:2/18:2/23:0)	-2.81	down
$(M+H)^+$	DG(4:0/20:2)	-2.6805	down
$(M+NH_4)^+$	TG(18:3/18:2/18:3)	-2.4242	down
$(M+NH_4)^+$	TG(18:2/18:2/16:2)	-2.191	down
$(M+NH_4)^+$	TG(18:2/18:2/18:3)	-2.159	down
$(M+NH_4)^+$	TG(25:0/18:2/18:2)	-2.1496	down
$(M+NH_4)^+$	TG(15:0/18:2/18:3)	-2.1198	down
$(M+H)^+$	PC(16:0/22:6)	-2.0475	down
$(M+NH_4)^+$	TG(16:0/16:1/21:4)	-1.9527	down
$(M+NH_4)^+$	TG(24:3/12:0/21:4)	-1.9462	down
$(M+NH_4)^+$	TG(16:0/24:0/24:0)	-1.9044	down
$(M+NH_4)^+$	TG(18:2/17:2/18:2)	-1.7345	down

$(M+NH_4)^+$	TG(18:0/18:0/24:0)	-1.5581	down
$(M+H)^+$	PE(18:3/18:2)	-1.4722	down
$(M+H)^+$	PC(18:3/18:3)	-1.3545	down
$(M+H)^+$	CerG2(d13:0/15:1)	-1.3321	down
$(M+H)^+$	PS(18:2/18:2)	-1.316	down
$(M+H)^+$	MG(26:6)	-1.3044	down
$(M+H)^+$	PC(17:0/20:4)	-1.2644	down
$(M+NH_4)^+$	TG(18:0/24:0/24:0)	-1.2099	down
$(M+NH_4)^+$	TG(26:0/18:0/18:1)	1.0903	up
$(M+NH_4)^+$	TG(18:0/17:0/18:1)	1.2924	up
$(M+NH_4)^+$	TG(19:1/12:0/17:1)	1.4815	up
$(M+NH_4)^+$	TG(19:3/19:5/24:6)	1.4815	up
$(M+NH_4)^+$	TG(6:0/20:5/21:4)	1.595	up
$(M+H)^+$	CerG1(d18:1/15:1)	2.0074	up
$(M+NH_4)^+$	TG(20:1/12:0/23:0)	2.0427	up
$(M+NH_4)^+$	TG(15:2/20:5/20:5)	5.4771	up
$(M+NH_4)^+$	TG(30:6/14:1/16:2)	5.7494	up
$(M+NH_4)^+$	PI(24:0/18:3)	18.081	up
$(M+NH_4)^+$	TG(15:0/8:0/16:0)	18.199	up
$(M-H)^-$	dMePE(18:0p/22:5)	-2.5161	down
$(M-H)^-$	LdMePE(38:4)	-2.3366	down
$(M-H)^-$	MGMG(10:3)	-1.9098	down
$(M-H)^-$	PS(18:2/18:2)	-1.4357	down
$(M+HCOO)^-$	PC(18:3/18:3)	-1.3503	down
$(M-H)^-$	LPE(16:0)	-1.2541	down
$(M-H)^-$	SQDG(27:4/27:4)	-1.2374	down
$(M-H)^-$	dMePE(14:0/18:3)	-1.0512	down
$(M+HCOO)^-$	PC(16:2/18:2)	-1.0149	down
$(M-H)^-$	MGDG(16:1/18:5)	1.0114	up
$(M-H)^-$	CerG1(d17:0/15:0)	1.1964	up
$(M+HCOO)^-$	PC(20:0/18:2)	1.1966	up
$(M-H)^-$	Cer(d25:0/10:0)	2.1248	up
$(M+HCOO)^-$	SQMG(7:1)	4.5719	up
$(M+HCOO)^-$	DGMG(1:0)	13.047	up
$(M-H)^-$	Cer(d25:0/22:3)	19.007	up

Supplementary Table S3. Significantly down-regulated lipids in a CV dose-dependent manner.

Extract mass	Lipid	f.value	p.value	FDR	Fisher's LSD
$(M+NH_4)^+$	TG(18:3/18:2/18:3)	355.66	2.30E-13	4.51E-11	0 - 100; 0 - 200; 100 - 200
$(M+NH_4)^+$	TG(18:2/18:2/16:2)	352.39	2.46E-13	4.51E-11	0 - 100; 0 - 200; 100 - 200
$(M+NH_4)^+$	TG(18:2/17:2/18:2)	242.96	3.74E-12	4.56E-10	0 - 100; 0 - 200; 100 - 200
$(M+NH_4)^+$	TG(18:2/18:2/18:3)	224.15	6.71E-12	6.14E-10	0 - 100; 0 - 200; 100 - 200
$(M+NH_4)^+$	TG(25:0/18:2/18:2)	153.55	1.03E-10	7.50E-09	0 - 100; 0 - 200; 100 - 200
$(M+NH_4)^+$	TG(15:0/18:2/18:3)	131.09	3.16E-10	1.93E-08	0 - 100; 0 - 200; 100 - 200
$(M+NH_4)^+$	TG(24:3/12:0/21:4)	103.55	1.67E-09	8.71E-08	0 - 100; 0 - 200; 100 - 200
$(M+NH_4)^+$	dMePE(18:2/13:0)	20.948	4.55E-05	0.0012726	0 - 100; 0 - 200; 100 - 200
$(M+NH_4)^+$	PE(18:2/15:0)	18.738	8.34E-05	0.0018965	0 - 100; 0 - 200; 100 - 200
